# Morphology of Peeled Internal Limiting Membrane in Macular Hole Surgery

**DOI:** 10.1155/2019/1345683

**Published:** 2019-05-02

**Authors:** Mun Y. Faria, David C. Sousa, Bruna C. Almeida, Andreia L. Pinto, Nuno P. Ferreira

**Affiliations:** ^1^Ophthalmology University Clinic, Faculdade de Medicina Lisboa, Universidade de Lisboa, 1649-028 Lisboa, Portugal; ^2^Ophthalmology Department, Hospital Santa Maria, Centro Hospitalar Universitário Lisboa Norte, 1649-035 Lisboa, Portugal; ^3^Histology and Comparative Pathology Laboratory, Instituto de Medicina Molecular, Lisboa, Portugal

## Abstract

**Purpose:**

The aim of this work was to describe the ultrastructure and behavior of peeled internal limiting membrane (ILM) in macular hole (MH) surgery.

**Methods:**

Seven patients with MH were included, and vitrectomy with ILM peeling was performed in all patients. The ILM inverted flap technique was used. Two other flaps of ILM of the same patient were collected and studied using light and transmission electron microscopy (TEM). ILM cell type, distribution, and morphology were analyzed, and the proliferation or fusion potential of the ILM interface was evaluated.

**Results:**

ILM vitreous sides in apposition showed signs of proliferative fibrotic activity, producing a basal membrane that merges ILM sides.

**Conclusions:**

Epiretinal cells in ILM show proliferative capacity, with formation of microfibrils between adjacent sides of the ILM, which may explain adherence of ILM flaps to the hole border, contributing to closure of the hole in MH surgery. This trail is registered with NCT03799575.

## 1. Introduction

Full thickness macular hole (MH) is an anatomic opening in the fovea, the central area of the retina, and affects mostly women after the 5th decade. As the fovea is responsible for central vision, the loss of vision caused by a MH is very severe. MH was considered untreatable until Kelly and Wendel reported closing a macular hole after pars plana vitrectomy, which removed all anteroposterior (AP) traction [1].

Besides the importance of AP vitreoretinal traction in MH formation, tangential traction seems to have a significant role in progression and recurrence of MHs [2]. Activated glial cells, especially Muller cells and astrocytes, may proliferate and migrate from the retinal side to the vitreous side and form epiretinal cells causing tangential traction [3]. Also, hyalocytes from the vitreous cavity may induce cellular proliferation at the internal limiting membrane (ILM) [4]. These hyalocytes are found on the vitreous cortex, in close contact with the ILM at the posterior retina, and may have macrophagic-like activity [2].

**Figure 1:**
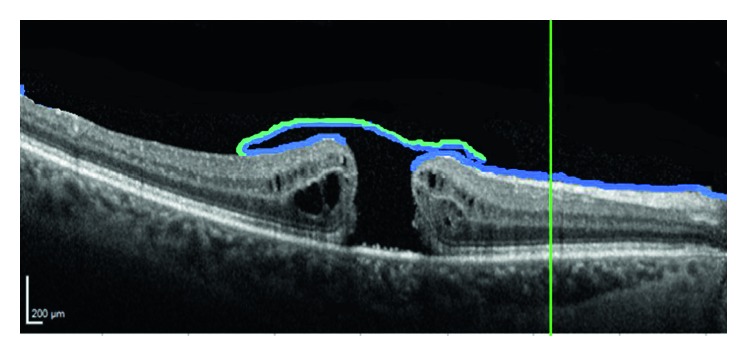
Schematic representation of the position of peeled ILM in macular hole surgery. Inverted flap with vitreous side adherent to vitreous side of ILM of the other border, not inverted.

ILM peeling is widely accepted as a safe surgical technique, showing a high success rate in MH closure [5]. The recent inverted flap technique introduced by Michalewska and Nawrocki for large macular holes allows for an even higher closure rate [6]. Instead of removing one piece of ILM, with this technique, the ILM is peeled until the hole border and one larger piece is left free and inverted over the hole and kept secure with intraocular gas, allowing for a large macular hole to close (Figure 1). The inverted flap to cover a large macular hole may be a temporal or superior flap of partially peeled ILM [7].

Herein, we describe novel findings concerning the morphological features of peeled ILM during IMH surgery that may help to explain the mechanisms of hole closure after MH surgery.

## 2. Patients and Methods

### 2.1. Surgical Procedure

Seven patients with MH larger than 400 *μ*m, according to OCT-based classification [8] were submitted to a standard surgical procedure. The surgery was performed by the same surgeon (MF) and consisted of a 23-gauge, three-port pars plana vitrectomy and ILM peeling. Balanced salt solution (BSS; Alcon, Fort Worth, TX) was used as an irrigation solution. Posterior vitreous detachment was completed when needed and assisted with triamcinolone acetonide. A single-use macular contact lens (Grieshaber®, Alcon, TX) was used in ILM peeling. Brilliant Blue® Dual (Geuder, Germany) assisted ILM identification, which was then engaged with an end-grip intraocular forceps. ILM was peeled in a rosette way around the macula and trimmed until the border of the hole, but one large flap was left, large enough to invert over the macular hole. Two other samples of ILM, per patient, were also collected, elsewhere in the macular area, and harvested for laboratory analysis.

The tenets of the Declaration of Helsinki were followed. All patients provided written informed consent to the surgical and study procedures. Approval was obtained from the Ethics Committee of Hospital Santa Maria.

### 2.2. Laboratory Analysis

Of the two samples of ILM per patient that were harvested, one was immediately fixed and submitted to optic microscopy (OM) and transmission electron microscopy (TEM) analysis and the other sample was incubated in enriched medium 199 (Gibco) for 20 minutes at room temperature, after which it was also fixed and submitted to OM and TEM analysis. Analysis of all samples followed the protocol available at https://doi.org/10.17504/protocols.io.qjiduke.

### 2.3. Image Acquisition

Six electron micrographs were acquired for each fragment, using a Hitachi H-7000 electron microscope equipped with a megaview III digital camera. Fields of interest were randomly selected, and 15,000x magnification images were acquired.

### 2.4. Quantitative Analysis

Collagen areas were measured using the iTEM software (Olympus©) measurement tool. For each micrograph, collagen areas were manually assessed in relation to the total amount of sample present, and average values for each patient and fragment were calculated. A collagen fraction (%) for each fragment was also calculated (values in *μ*m^2^).

## 3. Results

### 3.1. Macular Hole Closure

**Figure 2:**
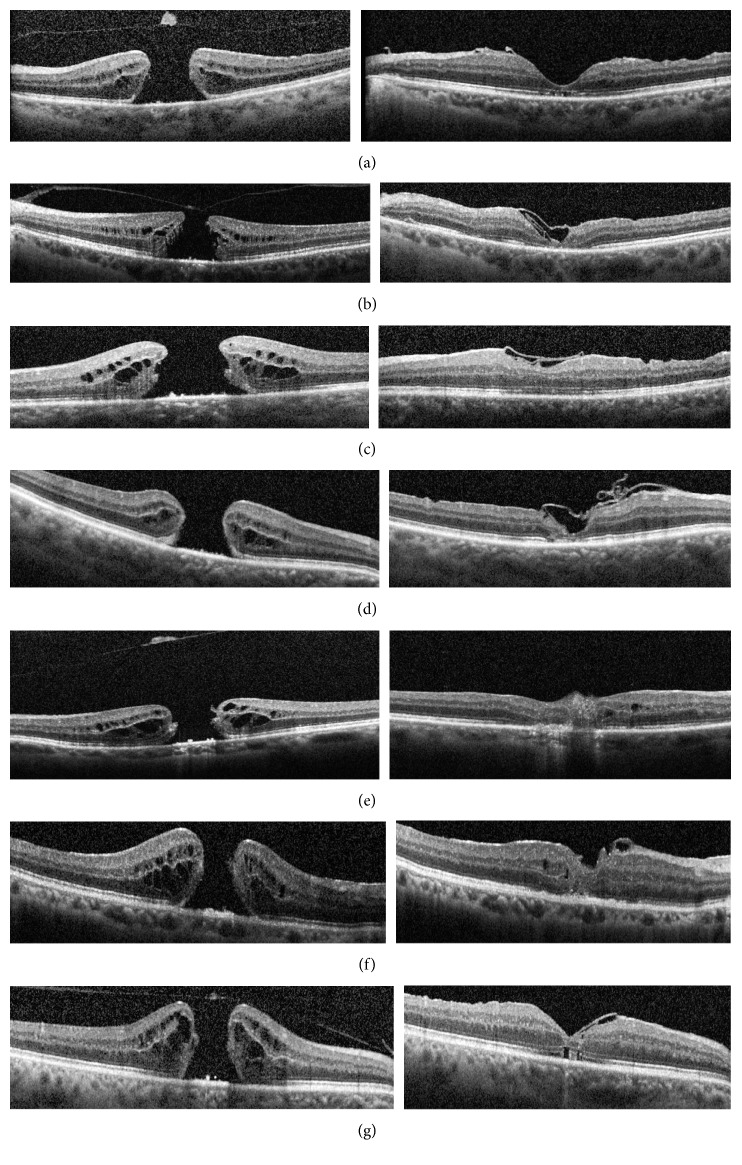
Preoperative (left) and postoperative (right) OCT of every patient. (a) Patient A. (b) Patient B. (c) Patient C. (d) Patient D. (e) Patient E. (f) Patient F. (g) Patient G.

The macular hole was closed and vision improved in all seven patients. In postoperative OCT, each macular hole was covered with ILM and bridging of external limiting membrane (Figure 2).

### 3.2. Histology and Immunohistochemistry

**Figure 3:**
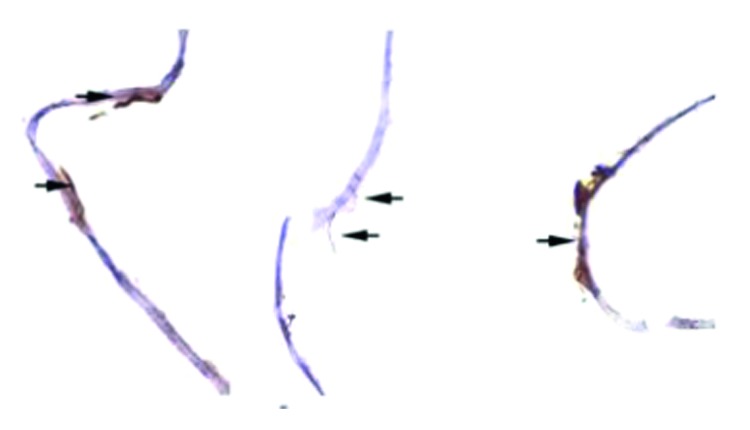
Internal limiting membrane (blue). Glial cells protein A coupled to 15 nm gold particle (gold), marked with black arrows and magnified.

ILM samples were stained with anti-GFAP antibody (antiglial fibrillary acidic protein), and the majority of cells found were positive for anti-GFAP. GFAP is the hallmark protein in astrocytes [9], a main type of glial cells in the central nervous system (Figure 3).

### 3.3. Light Microscopic Features

**Figure 4:**
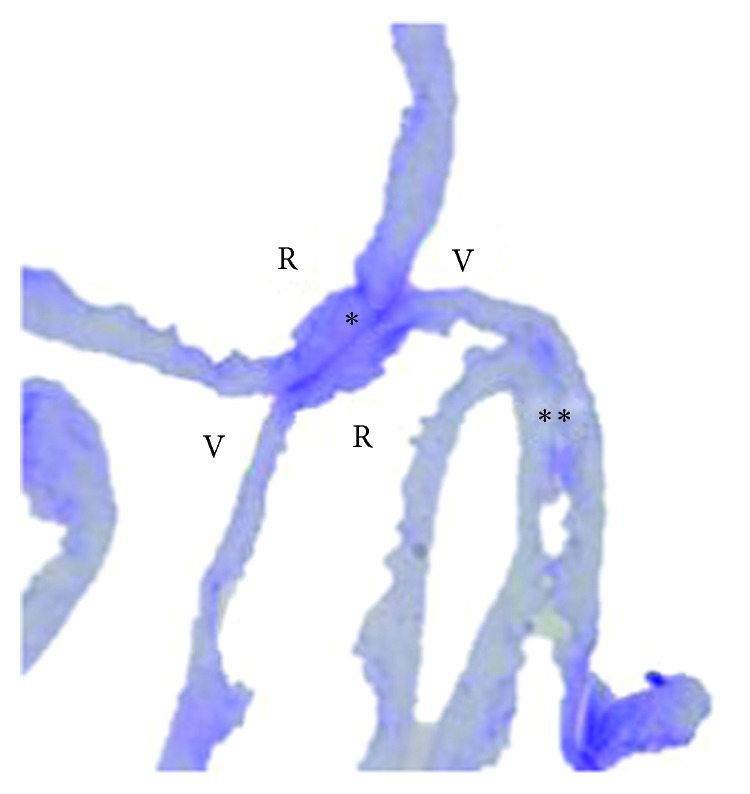
Optic light microscopy of ILM. V: vitreous side; R: retinal side. ^*∗*^Vitreous side contact; ^*∗∗*^retinal side contact.

Microscopy analysis of semithin sections of both samples showed a continuous folded and wrinkled ILM strand. The vitreous side of the ILM was smooth and continuous while the retinal side was characterized by irregular undulations (Figure 4).

### 3.4. Transmission Electron Microscopy

The morphological features of both the vitreous and the retinal sides of the ILM were evaluated in terms of cell distribution. The retinal side had scarce cells, while the vitreous side had some epiretinal cells.

**Figure 5:**
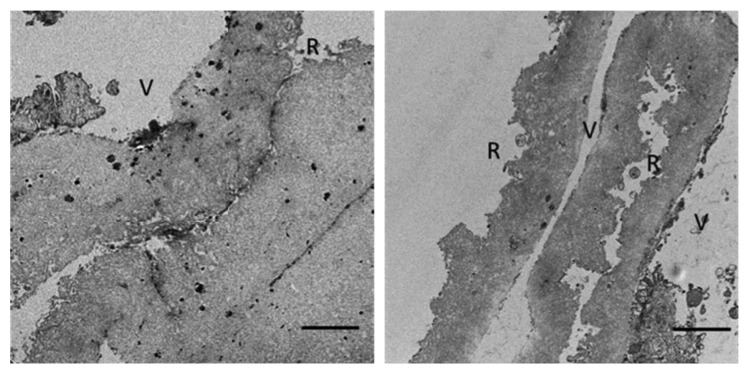
Juxtaposed sides of ILM with no apparent interaction between the vitreous side (V) and the retinal side (R). Bar: 2 *μ*m.

The same structures on both sides of the ILM were identified: the fragment that was immediately fixed was wrinkled with both the vitreous and retinal sides showing no evidence of merging activity nor fibrotic material between the two sides of the juxtaposed ILM (Figure 5).

**Figure 6:**
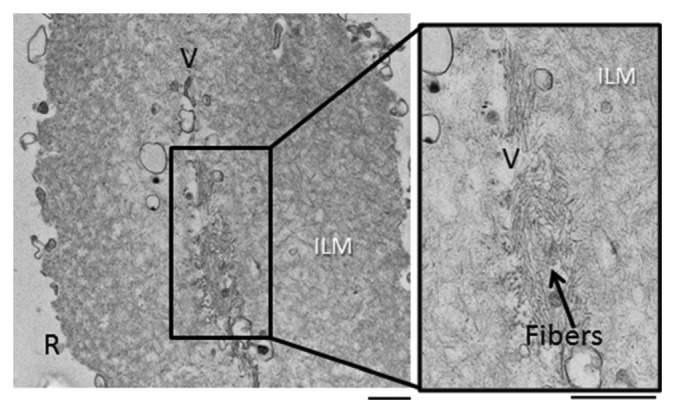
Representative electron micrographs of ILM of patient A, showing both vitreous sides (V) of the same membrane merged, and, in the center, the existence of fibers. Bar: 500 nm.

**Figure 7:**
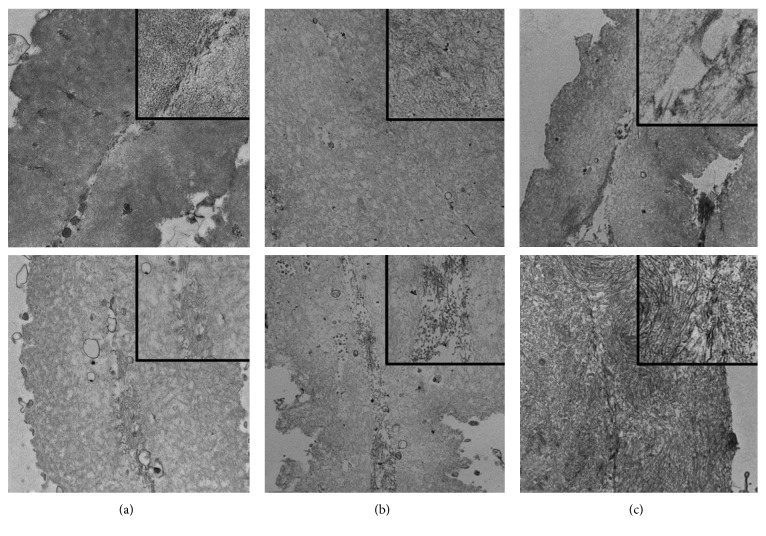
Transmission electron microscopy details of two internal limiting membrane (ILM) fragments from patients A, B, and C, with and without enriched medium. (a1–c1) Fragments of ILM from each patient which were immediately fixed. (a2–c2) A second ILM fragment of the same patient after 20-minute incubation in the enriched medium. Percentage area of collagen.

On the contrary, in the ILM sample that was kept in enriched medium for 20 minutes, the two vitreous sides of the folded piece of ILM came in contact and proliferative fibrotic material was present in the areas where adhesion occurred. In terms of morphology and structure, these fibers resemble collagen microfibrils/fibers (Figures 6 and 7).

Table 1 describes the percentage area of collagen in each observed sample, fixed immediately upon collection and after 20 min incubation with enriched medium.

## 4. Discussion

ILM peeling has been the standard procedure in MH surgery allowing closure rates of nearly 100% [10]. However, large macular holes, over 400 *μ*m diameter, have an increased risk of failure, and 44% do not close at first surgery, with 19% having been reported to stay flat and open [6]. Macular hole surgery has improved the rate of closure after the introduction of the inverted flap technique, especially in long-standing and large macular holes and in holes seen in high myopia [11]. Also, in cases of refractory macular holes, the autologous transplantation of ILM allowed for an improved anatomical outcome of these macular holes [12].

In these difficult macular holes, after releasing all anteroposterior and tangential tractions, the surgical technique of sealing the macular hole with inverted ILM consists in closing the hole with ILM peeled to the hole border, inverted over, and then attached to the borders of the hole, instead of completely peeling all ILMs around the hole. In case of autologous transplant, there is no more ILM near the hole to peel. A piece of ILM is peeled elsewhere in the retina of the same eye and is carefully placed on top of the hole, with the help of perfluorocarbon liquid, secured with air and gas. The peeled ILM, transplanted or inverted, contains Muller cells fragments that can induce gliosis on the retina and on the surface of the ILM. The macular hole closes, eventually due to the merging of the ILM with structures at the hole borders, and we speculate that the creation of this closed space may activate growth factors that induce cell realignment. In an experimental animal model, Shiode et al. tried to identify the components of the ILM that were important for the proliferation of Muller cells and collagen and fibronectin were found to enhance their migration [13]. Yokota et al. described newly synthesized collagen fibers in an ultrastructure study of peeled ILM after vitrectomy for myopic traction maculopathy [14]. Schumann et al. reported newly formed collagen at the vitreous side of the ILM removed from failed macular hole surgery [15]. In our study, we found a merging tendency of ILM pieces when kept in enriched media, in six of seven patients, accompanied by collagen fibers and fibrosis, as observed by TEM analysis. Considering our results and the results reported by Schumann at al. associated with the novel inverted flap, we speculate that this fibrosis may actually happen in the ILM vitreous side of our patients' eyes, allowing for hole closure, either because the vitreous side of the ILM has epiretinal cells or because of the presence of collagen fibers from the vitreous cavity.

In a clinical situation, surgery with vitrectomy and ILM peeling relieves anteroposterior and tangential mechanical forces. Gas tamponade creates prolonged contact of ILM tissue fragment with each other or with the underlying retinal tissue, which seems to be fundamental for hole closure. In our study, we found that when the two vitreous sides of ILM were in contact, epiretinal cells present in the ILM vitreous side form microfibrils that may contribute to the sealing process of MH surgery.

The limitations of this study include the small number of studied cases, with the possible consequence of randomness in the obtained findings. The identification of specific immunohistochemical markers for better cell characterization will also be a future added value. Also, the type of collagen, old and newly synthesized, has not been characterized.

## Figures and Tables

**Table 1:** Area of collagen in percentage (%) on samples fixed upon collection and after 20 min incubation with enriched medium.

	Collagen area fraction (%)	
	Fixed upon collection	Fixed after medium enrichment
Patient A	0.00	19.50
Patient B	0.00	6.68
Patient C	0.00	50.23
Patient D	5.77	17.78
Patient E	0.00	17.78
Patient F	0.00	0.00
Patient G	2.87	7.46

Values in %*μ*m^2^.

## Data Availability

All data used to support the findings of this study are available from the corresponding author upon request.
